# Toxicity evaluation of *Xanthorrhoea johnsonii* leaf methanolic extract using the *Artemia franciscana* bioassay

**DOI:** 10.4103/0973-1296.66929

**Published:** 2010

**Authors:** I. E. Cock, F. R. Kalt

**Affiliations:** 1 *Biomolecular and Physical Sciences, Nathan Campus, Griffith University 170 Kessels Road, Nathan, Queensland 4111 Australia*; 2 *Genomics Research Centre, Gold Coast Campus, Griffith University, Parklands Drive, Southport, Queensland 4222, Australia*

**Keywords:** Australian plants, grass tree, methanol extracts, toxic plants, *Xanthorrhoea johnsonii*

## Abstract

The toxicity of *Xanthorrhoea johnsonii* methanolic leaf extract was investigated using the *Artemia franciscana* nauplii bioassay. At 24 h, the extract produced an EC_50_ of 361.0 ± 41.8 *μ*g/ml, indicating that it was substantially more toxic than the pesticide Mevinphos (1346.2 ± 80.1 *μ*g/ml) and approximately 4 fold less toxic than potassium dichromate (87.1 ± 5.2 *μ*g/ml). Whilst potassium dichromate LC_50_ values remained constant across the 72-h test period, these values decreased for the extract and Mevinphos to similar values (199.8 ± 60.5 and 114 ± 12.8 *μ*g/ml, respectively), indicating their similar level of efficacy. Noteworthy was the apparent anesthetic effect of *X. johnsonii* leaf extract. Although the extract initially appeared to kill the *A. franciscana nauplii*, they were seen to temporarily recover by 48 h only to die by 72 h.

## INTRODUCTION

Plants produce a wide variety of compounds, which in addition to giving them characteristic pigment, odor and flavor characteristics, may also have medicinal and therapeutic properties.[1] For thousands of years, traditional plant-derived medicines have been used in most parts of the world and their use in fighting disease is becoming the focus of intense study.[2,3] Much of the research into traditional medicinal plant use has focused on Asian,[4] African[5] and South American[6] plants. Relatively few studies have focused on the medicinal properties of Australian native plants.

*Xanthorrhoea* (grasstree) is a small genus of evergreen plants consisting of 28 species and 5 subspecies, all of which are endemic to Australia.[7] All species are long-lived with some plants being estimated at more than 550 years of age.[7–9] However, *Xanthorrhoea* species are also extremely slow growing. Indeed, the growth of *Xanthorrhoea johnsonii* has been estimated to be at a rate of 0.88 cm/year.[9,10] Some species are stemless whilst others develop a thick, sometimes branching, trunk topped by a dense crown of rigid leaves. In species with a trunk, this trunk may take up to 20 years to develop.[11]

Australian Aboriginal people had many traditional uses for *Xanthorrhoea* species including use as tools and weapons[12–14] and as a resin.[12,15] *Xanthorrhoea* species also provided a minor food source for Aboriginals. Tubers[13] and shoots[12] were eaten whilst nectar from the flowers was used to make a sweet drink.[13] The seeds could also be ground to make dough. Since European settlement, *Xanthorrhoea* species were extensively harvested for their resin[7] until they were declared protected species.

Being such a slow growing group of plants, it is likely that *Xanthorrhoea* species have developed protective mechanisms to deter animal foraging which could potentially threaten their survival. Indeed, the list of animals that use *Xanthorrhoea* species as a major food source is relatively small.[7] Whilst several species of insects, birds and mammals are known to use *Xanthorrhoea* for food, they almost exclusively feed on the flowers and seeds, leaving all but the youngest leaves largely untouched. Indeed, *X. johnsonii* has been reported to poison cattle that graze on its leaves.[16–19] Cattle poisoned by *Xanthorrhoea* become uncoordinated and lose condition, become dehydrated, and in severe cases die. It is possible that a chemical protectant/deterrent produced by *Xanthorrhoea* species discourages foraging by herbivores, allowing juvenile plants to reach maturity.

This study was undertaken to examine the toxicity of *X. johnsonii* leaves using the *A. franciscana* nauplii (brine shrimp larvae) lethality bioassay. This assay has been used to examine the toxicity of a wide variety of compounds.[20] It is an efficient, inexpensive and a relatively rapid way to detect toxic compounds, requiring only low amounts of sample (<20 mg). This test correlates well with cytotoxic activity of some human tumors and, therefore, has the potential to detect new antitumour agents.[21]

## MATERIALS AND METHODS

### Plant collection and extraction

*X. johnsonii* is one of the two species of *Xanthorrhoea* occurring naturally in Toohey Forest, Brisbane, Australia (along with *Xanthorrhoea macronema*) and is by far the dominant species. *X. johnsonii* leaves were collected from Toohey Forest and were identified with reference to a taxonomic key to Toohey Forest plants.[22] Samples were dried in a Sunbeam food dehydrator and then ground to a coarse powder. One gram of the powdered leaves was extracted extensively in 50 ml methanol (Ajax, AR grade) for 24 h at 4°C with gentle shaking. The extract was passed through filter paper (Whatman No. 54) under vacuum, followed by drying by rotary evaporation in an Eppendorf concentrator 5301. The resultant pellet was dissolved in 10 ml of 20% methanol. The extract was passed through 0.22 μm filter (Sarstedt) and stored at 4°C.

### Reference toxins for biological screening

Potassium dichromate (K_2_Cr_2_O_7_) (AR grade, Chem-Supply, Australia) was prepared as a 1.6 mg/ml solution in distilled water and was serially diluted in synthetic seawater (described below) for use in the *A. franciscana* nauplii bioassay. Mevinphos (2-methoxycarbonyl-1-methylvinyl dimethyl phosphate) was obtained from Sigma-Aldrich as a mixture of *cis* (76.6%) and *trans* (23.0%) isomers and prepared as a 4 mg/ml stock in distilled water. The stock was serially diluted in artificial seawater for use in the bioassay.

### *A. franciscana* nauplii toxicity screening

Toxicity was tested using the *A. franciscana* nauplii lethality assay developed by Meyer *et al*,[20] for the screening of active plant constituents, with the following modifications. *A. franciscana* cysts were obtained from North American Brine Shrimp, LLC, USA (harvested from the Great Salt Lake, Utah). Synthetic seawater was prepared using Reef Salt, AZOO Co., USA. Seawater solutions at 34 g/l distilled water were prepared prior to use. Two grams of *A. franciscana* cysts were incubated in 1 l synthetic seawater under artificial light (2000 Lux) at 25 °C with continuous aeration. Hatching commenced within 16–18 h of incubation. Newly hatched *A. franciscana* (nauplii) were used within 10 h of hatching. Nauplii were separated from the shells and remaining cysts and were concentrated to a suitable density by placing an artificial light at one end of their incubation vessel and the nauplii-rich water closest to the light was removed for biological assays. Four hundred microliters of seawater containing approximately 50 (mean 53, *n* = 168, SD 12) nauplii was added to wells of a 48-well plate and immediately used for bioassay. The plant extract was diluted to 2 mg/ml in seawater for toxicity testing, resulting in a 1 mg/ml concentration in the bioassay. Four hundred microliters of diluted plant extract and the reference toxins were transferred to the wells and incubated at 25 ± 1 °C under artificial light (1000 Lux). A negative control (400 μl seawater) was run in at least triplicate for each plate. All the treatments were performed in at least triplicate. The wells were checked at regular intervals and the number of dead counted. The nauplii were considered moribund if no movement of the appendages was observed within 10 s. After 72 h, all nauplii were sacrificed and counted to determine the total number per well. The LC_50_ with 95% confidence limits for each treatment was calculated using probit analysis.[23]

## RESULTS AND DISCUSSION

**Figure 1:**
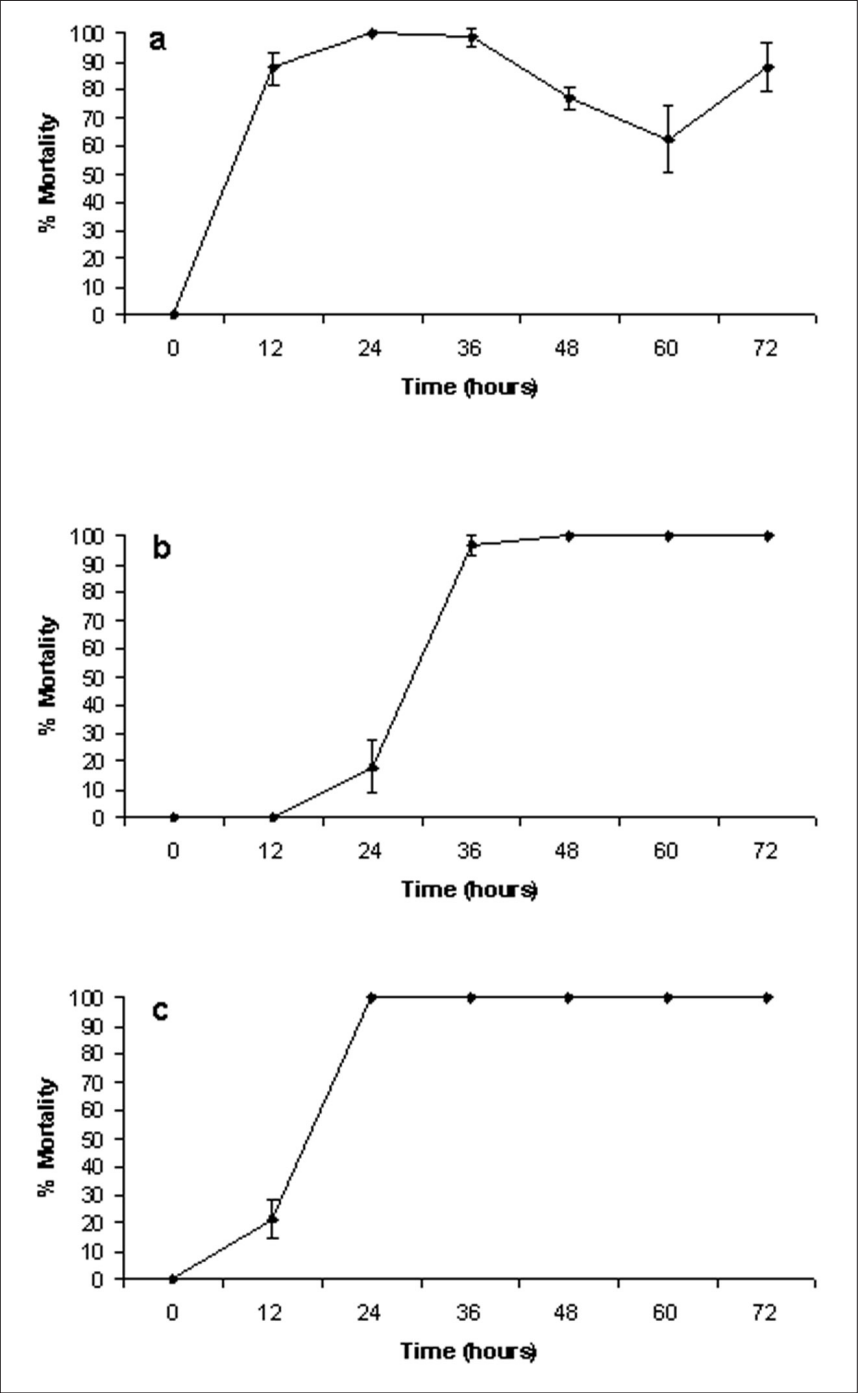
Brine shrimp lethality of (a) *X. johnsonii* extract (1000 µg/ml), (b) Mevinphos (2000 µg/ml), (c) potassium dichromate (800 µg/ml). All bioassays were performed in at least triplicate and are expressed as mean ± standard deviation

One gram of powdered dried *X. johnsonii* leaves was extensively extracted with methanol and dried under vacuum, resulting in 39 mg of dried extracted material. Resuspension of the dried fraction in 10 ml of 10% methanol resulted in the crude test extract concentration of 3.9 mg/ml. The extract was diluted to 2000 μg/ml in artificial seawaterfor toxicity testing, resulting in a 1000 μg/ml concentration in the *A. franciscana* lethality bioassay. *A. franciscana* nauplii were also tested against methanol dilutions to determine the effect of residual methanol on toxicity. No increase in mortality above the control values was seen when testing methanol concentrations up to 5% in the assay, indicating that the residual methanol in the re-suspended extracts was not responsible for nauplii mortality (unpublished results). The results of *A. franciscana* bioassay screening of the *X. johnsonii* methanolic extract and the control toxins are shown in Figure 1.

Two interesting features of *X. johnsonii* toxicity were noted. Firstly, *X. johnsonii* extract induced apparent morbundity much more rapidly than was observed for either of the positive control toxins. High levels of apparent morbundity (87.6 ± 5.8%) were seen with *X. johnsonii* extract after 12-h exposure, with 100% mortality seen by 24 h. In contrast, both Mevinphos and potassium dichromate took much longer to achieve high levels of mortality. Following 12 h exposure, both Mevinphos and potassium dichromate had only induced low levels of mortality (0 and 21.3 ± 6.9%, respectively). Indeed, 36 h was required by Mevinphos to achieve approximately 100% mortality (96.9 ± 3.3%).

Perhaps more interesting was the decrease in apparent morbundity seen between 36 and 60 h of exposure. Throughout the course of these studies and previous studies within this laboratory, lack of movement of *A. franciscana* appendages for 10 s was considered as an indication of apparent morbundity. Many other studies have been less rigorous in their definition of apparent morbundity when determining LC_50_ /EC_50_ values. Several studies have defined death (for LC_50_ determination) as the lack of controlled forward movement. As this could indicate intoxication from which the brine shrimp could recover, we have defined a total lack of movement, over a period of 10 s, as our endpoint. Surprisingly, many of the *A. franciscana* nauplii used in these studies recovered from their apparent “death” and were seen to swim normally by 48 h exposure. These studies have been repeated several times using a longer time period (10 min) for lack of movement of appendages as a definition of death and the same trend is evident. It appears that the *X. johnsonii* extract has an anesthetic affect similar to the effect previously reported for curare,[24–26] from which the *A. franciscana* nauplii are able to temporarily recover. However, at 60 h, it became evident that the nauplii were experiencing difficulty in swimming (“strobe-like” swimming). Following 60 h, the percentage apparent moribundity again began to increase. The nauplii did not recover from this moribund state a second time.

**Figure 2:**
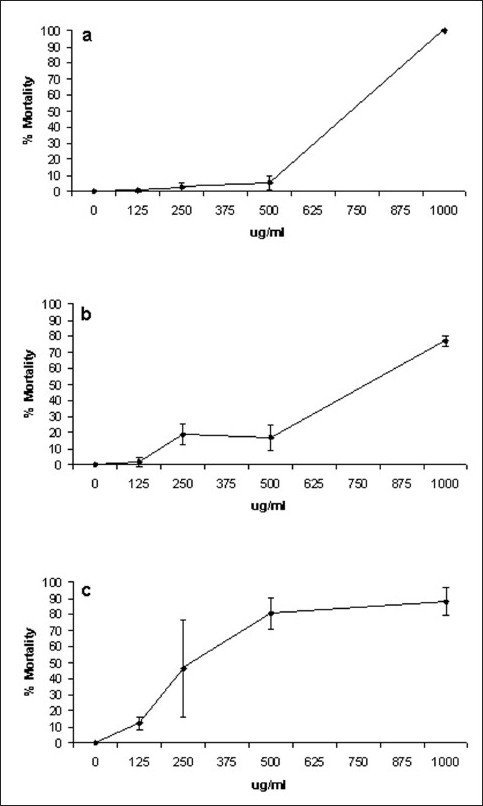
Dependence of *X. johnsonii* extract concentration on *A. franciscana* apparent moribundity following exposure for (a) 24 h, (b) 48 h, (c) 72 h. *X. johnsonii* extract was diluted freshly prior to use in deionized water; 400 µl of juice dilution was added to 400 µl of saline containing *A. franciscana nauplii*. Moribundity was defined as the lack of appendage movement for at least 10 s. All the bioassays were performed in at least triplicate

**Figure 3:**
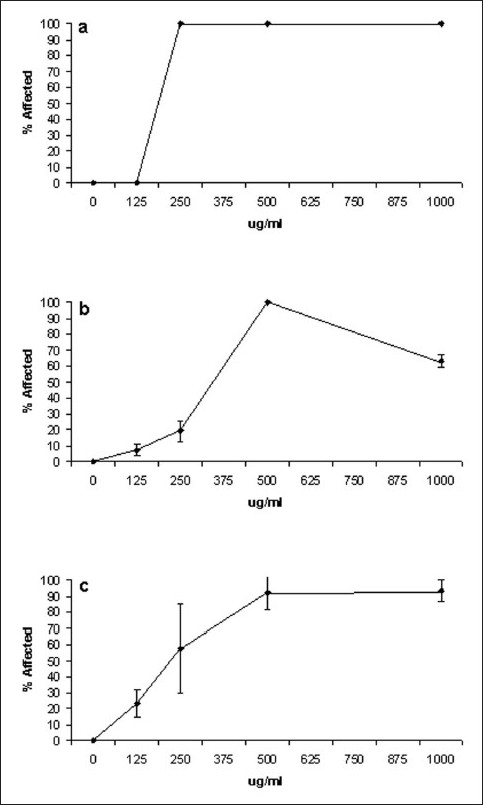
Dependence of *X. johnsonii* extract concentration on percentage of affected *A. franciscana*, following exposure for (a) 24 h, (b) 48 h, (c) 72 h. *X. johnsonii* extract was diluted freshly prior to use in deionized water; 400 µl of juice dilution was added to 400 µl of saline containing *A. franciscana nauplii*. Affected nauplii were defined as the lack of controlled forward movement. All bioassays were performed in at least triplicate

A concentration–response analysis was undertaken to determine the dependence of percentage apparent moribundity on *X. johnsonii* extract concentration. Due to the previously described decrease in moribundity with time, we report both on the percentage dead (as defined by lack of movement of appendages for 10 s) [Figure 2] and percentage affected (lack of controlled forward movement) [Figure 3]. The same trend previously described for 1000 μg/ml *X. johnsonii* extract was also apparent in these concentration-response studies. Apparent moribundity of *A. franciscana* exposed to 1000 μg/ml *X. johnsonii* extract again decreased from 100% moribund at 24 h to 76.7% (±3.6) by 48 h and increased again to 87.9% (±8.5) by 72 h. The trend was even more apparent when percentage affected was monitored [Figure 3]. The percentage of affected *A. franciscana* exposed to 1000 μg/ml *X. johnsonii* extract decreased from 100% affected at 24 h to 63.2% (±3.9) by 48 h and increased again to 93.4% (±6.6) by 72 h.

LC_50_ values for the *X. johnsonii* extract, potassium dichromate and mevinphos were calculated by probit analysis [Table 1]. Similarly, EC_50_ values for *X. johnsonii* extract were also calculated. Both the extract and the control toxins displayed significant correlation between the concentration/dilution and the percentage affected. The R^2^ coefficients for the lines of regression of each test sample were greater than 0.9 with the response linear and statistically significant at *P* < 0.05.

**Table 1:** LC_50_ and EC_50_ determinations of *X. johnsonii* extract and the reference toxins Mevinphos and potassium dichromate; values indicate the mean of triplicate determinations ± standard deviation

Treatment	LC_50_ value in µg/ml (95% confidence interval) at time (h)		
	24 h	48 h	72 h
*X. johnsonii* extract	361.0 ± 41.8	402.1 ± 15.7	199.8 ± 60.5
*X. johnsonii* extract*	220.1 ± 8.6*	250.0 ± 9.8*	187.5 ± 19.8*
Mevinphos	1346.2 ± 80.1	523.3 ± 39.0	114 ± 12.8
Potassium dichromate	87.1 ± 5.2	81.9 ± 3.8	79.6 ± 4.6

* Indicates EC_50_ determinations (where effect is defined as lack of controlled forward movement)

## CONCLUSIONS

The results reported here demonstrate the toxicity of methanolic extracts of *X. johnsonii* leaves, providing a possible explanation for the lack of use of the leaves as a food source for Australian native herbivores. Moreover, as the *A. franciscana* nauplii bioassay used in these studies correlates well with antitumor activity,[21] it is possible that *X. johnsonii* leaf extracts may also be effective as anti-tumor agents. Further studies are needed to determine whether this is the case and to identify the factors involved and their mechanism of action.

Perhaps more interesting was the apparent anesthetic effect of the methanol extract on the *A. franciscana* nauplii. This effect is similar to the effects previously described for tubocurarine, dimethyltubocurarine and alcuronium (collectively known as curare, a South American arrow poison) from *Chondrodendron tomentosum*.[24–26] Curare is a neuromuscular blocker that was commonly used as a muscle relaxant by anesthesiologists prior to the development of safer, more effective muscle relaxants. Curare functions by blocking neuronal nicotinic acetylcholine receptors (nAChR),[27] thus blocking neurotransmission. Determining whether the *X. johnsonii* leaf methanolic extract functions by a similar mechanism or not, was outside the scope of this study. Further studies are needed to isolate individual components from *X. johnsonii* leaf and examine their mechanism/s of action. It is possible that novel anesthetics/muscle relaxants may be discovered by such studies.
